# Characterization of the Na^+^/H^+^ Antiporter from *Yersinia pestis*

**DOI:** 10.1371/journal.pone.0026115

**Published:** 2011-11-15

**Authors:** Assaf Ganoth, Raphael Alhadeff, Dovrat Kohen, Isaiah T. Arkin

**Affiliations:** Department of Biological Chemistry, The Alexander Silberman Institute of Life Sciences, The Hebrew University of Jerusalem, Jerusalem, Israel; George Mason University, United States of America

## Abstract

*Yersinia pestis*, the bacterium that historically accounts for the Black Death epidemics, has nowadays gained new attention as a possible biological warfare agent. In this study, its Na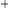/H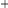 antiporter is investigated for the first time, by a combination of experimental and computational methodologies. We determined the protein's substrate specificity and pH dependence by fluorescence measurements in everted membrane vesicles. Subsequently, we constructed a model of the protein's structure and validated the model using molecular dynamics simulations. Taken together, better understanding of the *Yersinia pestis* Na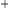/H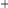 antiporter's structure-function relationship may assist in studies on ion transport, mechanism of action and designing specific blockers of Na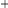/H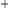 antiporter to help in fighting *Yersinia pestis* -associated infections. We hope that our model will prove useful both from mechanistic and pharmaceutical perspectives.

## Introduction

Na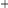/H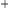 antiporting, already postulated in 1966 [1], and confirmed experimentally in 1974 [2], is a key determinant in the homeostasis of pH and [Na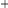], the latter having an additional impact on cell volume as well. Na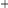/H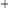 antiporters can be found ubiquitously in plants, animals and microorganisms, and are present in cell plasma membranes and in the membranes of many eukaryotic organelles [3]. The best characterized member of this bacterial family of proteins is the Na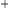/H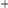 antiporter A, NhaA, present throughout the bacterial domain. NhaA members were reported to be selective for Na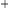 and Li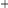 [4] allowing them to detoxify the cell in case of Li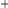 poisoning [5], and in other cases for K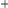 as well [6].

Currently, the only crystal structure obtained for the Na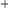/H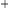 antiporter family is the one of *Escherichia coli* NhaA, solved to 3.45 Å [7]. The structure reveals twelve transmembrane segments (TMSs) that are connected by outer-membrane loops. TMSs IV and XI are crossed and form an assembly made of two oppositely oriented 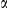-helices, each perturbed in its middle by a short unfolded stretch. There are two vestibules; a negatively charged one starting at the middle of the membrane, near the putative ion-binding site (D164) and opening out to the cytoplasm, and a smaller narrower vestibule spanning from the middle of the membrane toward the periplasm. The vestibules converge from both sides of the membrane into the TMSs IV/XI assembly; bearing in mind the non-canonical arrangement of unstructured coils inside the membrane, an important role for this assembly is implied. At the periplasmic side of the protein, the loop between helices I and II contains a 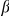-hairpin that forms together with the other loops a rigid periplasmic face parallel to the membrane. At its cytoplasmic side, many helices protrude into the cytoplasm forming a rough face.

The activity of the *Escherichia coli* NhaA is pH dependent; it is reduced by more than three orders of magnitude when pH is lowered from 8.5 to 6.5 [8]. This regulation is a common characteristic of many Na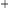/H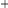 transporters and requires a “pH sensor”, which under different protonation states leads to conformational changes of the protein, affecting its activity [9], [10].

Previous mutation experiments showed that aspartic acid residues, which are located adjacent to the TMSs IV/XI assembly (D133, D163 and D164) are essential to NhaA's activity [11]. Recent molecular dynamics (MD) simulations on *Escherichia coli* NhaA have suggested a possible mechanism for the ion exchange [12]. According to the proposed mechanism, D164 serves as the Na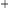 -binding site while D163 serves as the molecular “switch” between the conformations of the protein.

The *Yersinia pestis* bacterium accounts for three huge pandemics since the sixth century with millions of deaths (including the Black Death, one of the most devastating pandemics in human history [13]) as well as numerous smaller epidemics and sporadic cases [14]. It is a gram-negative, facultative anaerobic, rod-shaped bacterium belonging to the family *Enterobacteriaceae* that has been identified as the etiological agent of plague in humans and animals (for reviews see [15], [16]). Plague is still an endemic illness in many areas of the world, having potential devastating consequences [17]. *Yersinia pestis* is of special interest not only because of its illness causing ability but also due to its purposeful hazardous misuse for warfare purposes [18], being classified as a category A of potential biological weapon by the US Centers for Disease Control. Although the high clinical and pharmaceutical interest of *Yersinia pestis*, its major salt pump, the Na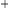/H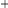 antiporter has never been investigated and its structure, substrate selectivity and pH dependence are yet to be determined. Therefore, this work addresses these issues.

The current study involves both experimental and computational analyses, in a synergistic manner, aimed to characterize the Na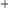/H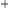 antiporter from *Yersinia pestis*. To account for the experimental part of this work, we first showed that *Yersinia pestis* NhaA confers full complementation to *Escherichia coli* bacteria lacking antiporter systems, otherwise unable to grow at the presence of Na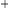 or Li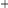. We then used vesicles containing the NhaA protein, and followed fluorescence quenching to determine the protein's ion selectivity and pH profile. Secondly, based on the *Escherichia coli* NhaA crystal structure, we constructed an initial model for the *Yersinia pestis* NhaA protein's structure by means of homology modeling procedure. Then, we evaluated the quality of the structure by performing a series of atomic-level MD simulations and subsequent analyses. Taken together, we propose a prototype blueprint model structure and a biochemical characterization for the *Yersinia pestis* NhaA that can be considered as working tools to aid subsequent theoretical and experimental studies.

## Results and Discussion

The goal of our work was characteriz ing the major salt pump from *Yersinia pestis*. Towards that end we cloned the NhaA homologue from *Yersinia pestis* and experimentally characterized its complementarity in *Escherichia coli*, ion selectivity and pH activity profile. Subsequently, we computationally examined the protein in detail using homology modeling and molecular dynamics simulations.

### Experimental analyses

#### Cloning and functionality

**Figure 1:**
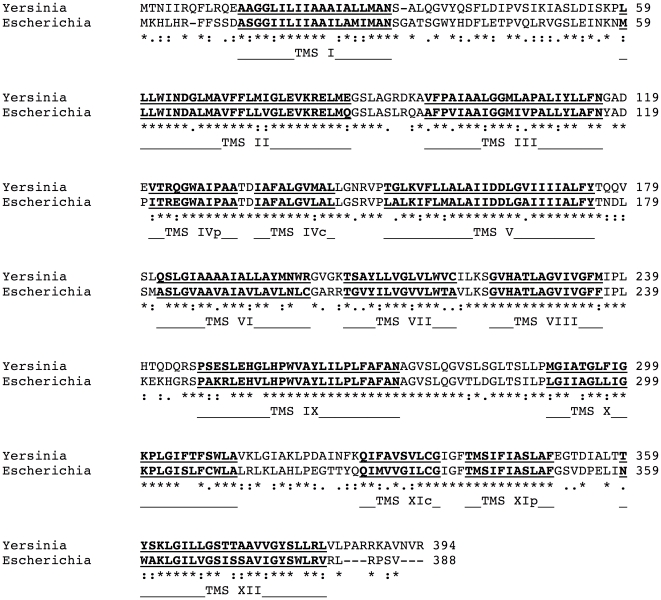
BLAST sequence alignment between *Escherichia coli* NhaA (PDB entry 1ZCD) and *Yersinia pestis* NhaA (Entrez accession code NP_994981). Stars, colons and dots refer to identical residues, conserved and semi-conserved substitutions, respectively. TMSs are sequentially indicated.

As stated above, in *Escherichia coli* NhaA is the major Na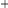 pump responsible for salinity and pH homeostasis. We therefore used the sequence of Ec-NhaA to search for homologous proteins in the *Yersinia pestis* KIM D10 genome [19]. A single hit was found that is 67% identical to the *Escherichia coli* protein with an E value of 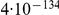 (see Figure 1). Furthermore, whole genome comparison did not reveal a *Yersinia pestis* homolog of the NhaB, NhaC and NhaD proteins.

**Figure 2:**
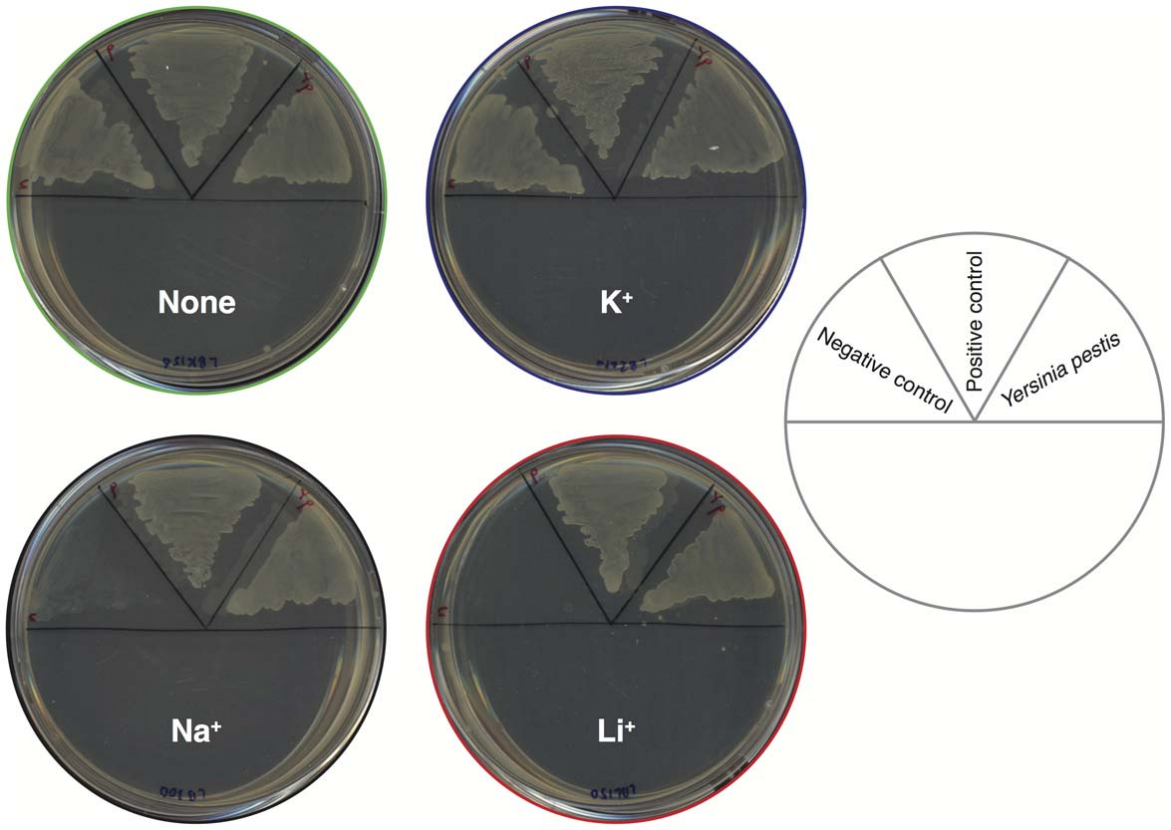
Na 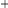
 **and Li** 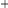 **complementation analysis of the** ***Yersinia pestis*** **NhaA.** *Yersinia pestis* NhaA was expressed in the KNabc bacterial strain that does not contain any native antiporter [20]. Subsequently, the protein was examined for its ability to allow growth of the bacteria in 300 mM [Na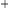 ] or 150 mM [Li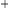 ], as indicated. Bacteria that did not harbor any antiporter were used as negative control, while expression of *Escherichia coli* NhaA was used as positive control.

Since the two proteins share extensive sequence resemblance, we sought to test the functionality of the *Yersinia pestis* NhaA, by performing an *in vivo* complementation assay. We tested the protein's ability to allow growth, in the presence of stressing Li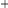 or Na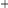 ions, of bacteria that do not harbor any other antiporters [20]. As shown in Figure 2, we have found that *Yersinia pestis* NhaA confers full rescue to *Escherichia coli* bacteria lacking antiporter systems, which are otherwise unable to grow on Na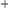 or Li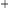. This indicates that the sub-cloned *Yersinia pestis* NhaA gene encodes and expresses an active and readily functional protein. To obtain further insight into its activity characteristics, we further tested the *Yersinia pestis* NhaA in *in vitro* analyses as described below.

#### Selectivity

**Figure 3:**
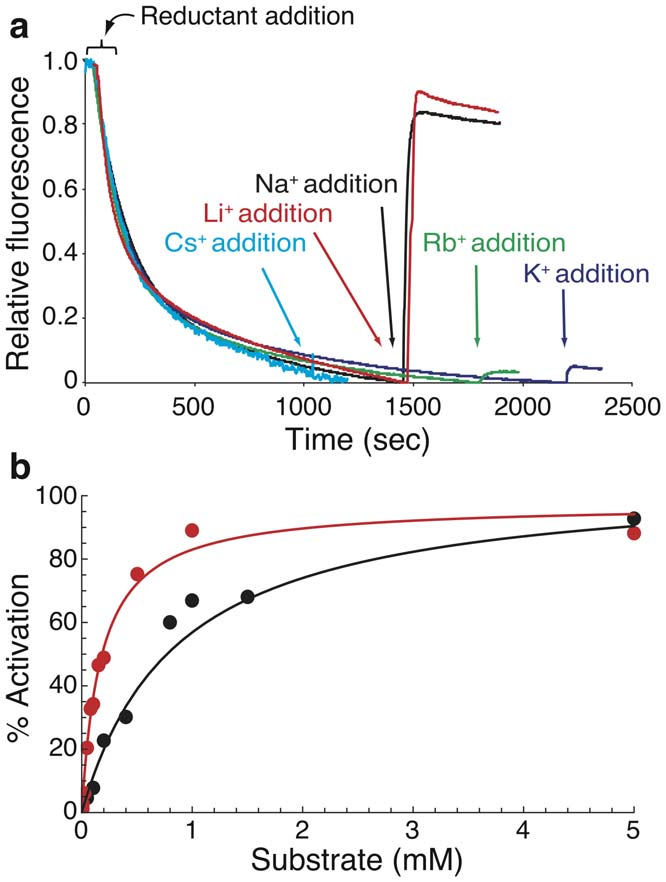
NhaA activity assay in everted membrane vesicles. Everted membrane vesicles activity was determined using acridine orange fluorescence to monitor 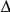
pH. (a) Data of typical measurements are shown. At the onset of the reaction, succinic acid (0.6 mM) was added to energize the vesicles and fluorescence was recorded until a steady state level of 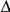
pH (100% quenching) was reached. Then, 5 mM of Na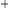 (black), Li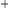 (red), K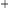 (purple) Rb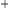 (green) or Cs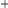 (magenta) were added to evoke the Na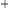/H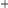 antiporter. NhaA activation level was defined as the percentage of dequenching at steady state after adding Na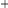 or Li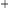 , from those 100%; (b) Michaelis-Menten kinetic fit. Activation of NhaA was measured at different Na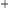 (black) and Li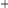 (red) concentrations. Data points (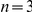) are shown in circles, while the fit is shown in solid lines.

In order to determine *in vitro* the protein's selectivity, we performed an activity assay examining different alkali ions as substrates (Figure 3 a). The activity was estimated from the change caused by each tested ion to the 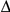
pH across the everted membrane as measured by the fluorescent probe acridine orange [21], [22]. After energizing the everted membranes with succinic acid, quenching of the fluorescence achieved a steady state level and then different ions were added to test their ability to activate the antiporter. To validate that the activity thrives from NhaA function, we tested the possibility of dequenching upon addition of Na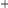 or Li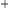 in the absence of NhaA. As expected, the fluorescence did not change when the antiporter was not present (see Figure S1). Conversely, when NhaA was present, upon addition of either Na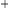 (black) or Li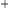 (red) we observed a profound dequenching, indicating a major activation of the protein. Addition of K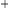 (purple) or Rb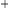 (green) caused slight dequenching, implying that the protein is not completely selective against them, whereas Cs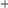 (magenta) did not evoke detectable dequenching at all. Hence, we report that the *Yersinia pestis* Na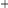/H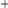 antiporter is selective for Na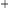 and Li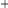, but shows some leakiness for other alkali ions. This observation is most striking considering their radii vary two-folds, suggesting that geometrical considerations are not the sole determinants that govern selectivity. In conclusion, the antiporter from *Yersinia pestis*, in terms of selectivity, may represent an intermediate form between the strictly selective *Escherichia coli* NhaA [4] and the K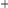 permissive antiporter from *Vibrio parahaemolyticus* [6].

Having established that the protein is mainly activated by Na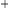 or Li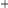, we proceeded to determine the activation kinetics by measuring the protein's pumping activity as a function of Na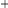 or Li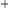 concentrations (Figure 3 b). We fit a Michaelis-Menten curve to our data (for both ions, the calculated R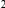 of the fit was above 0.96) and obtained the following kinetic values: the 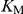 and 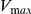 for Na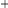 are 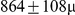
M and 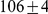 (a.u.) whereas for Li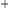 they are 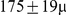
M and 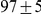 (a.u.).

Our calculated 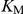 and 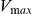 values indicate that: (i) The *Yersinia pestis* Na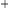/H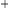 antiporter has about 4.5 times higher affinity to Li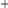 over Na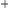. This is in accord with experimental [23], [24] and computational [12], [25] studies suggesting that the *Escherichia coli* Na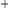/H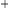 antiporter has a significant higher affinity for Li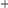 over Na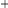; (ii) The affinity of the *Yersinia pestis* Na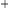/H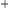 antiporter is higher than that of the *Escherichia coli* Na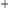/H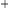 antiporter. This is reflected by lower 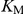 values for both ions measured at the former Bacterium over the latter [25]. Hence, the *Yersinia pestis* Na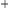/H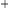 antiporter is a more potent and robust pump in terms of affinity, making it sensitive even to relatively low [Na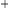 ] or [Li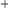 ]. Finally, comparison of the 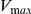 values obtained for Na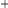 and Li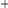 shows that they are indistinguishable (see Figure 3 b). Hence the pump's preference for Li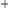 over Na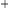 is solely a function of differential affinity and not turnover.

#### pH dependence

**Figure 4:**
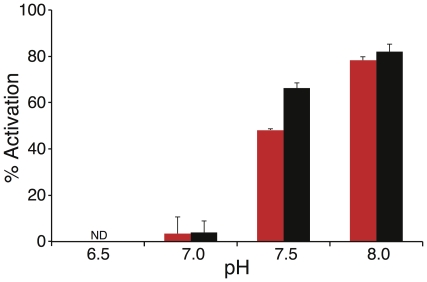
Effect of pH on the Na 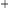
 **/H** 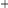 **antiporter activity in everted membrane vesicles.** Activity of the Na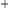/H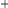 antiporter was estimated as described in Figure 3 at various pH values after addition of Na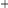 (1 mM, black) or Li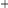 (0.2 mM, red). Percent of dequenching (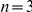) due to cation addition is depicted versus medium pH. Note that at pH 6.5 no activity was observed for both ions.

The activity of NhaA from *Escherichia coli* is highly pH dependent [8]. Hence we decided to examine the pH activity profile of the *Yersinia pestis* NhaA as well. We measured the protein's activity at different pH values (Figure 4) using the fluorescence quenching method using Na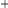 (black) or Li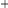 (red) as a substrate ion (the ions' concentrations used for activation were chosen to be slightly higher than the calculated 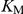 for each ion, using 1 mM for Na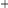 and 0.2 mM for Li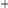). We have found that the *Yersinia pestis* Na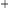/H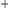 is active at a pH range similar to that of the *Escherichia coli* Na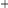/H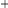 [8], exhibiting no detectable activity at pH 6.5, and an activity increase when pH is raised from 7 to 8. A similar trend was observed for both ions. Since pH regulation is a common characteristic of many Na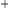/H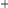 transporters, we conclude that the *Yersinia pestis* Na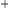/H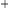 (like the Na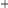/H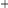 antiporter from *Escherichia coli* [10] and *Vibrio parahaemolyticus* [6], [26]), is pH dependent and harbors a “pH sensor”, that forces conformational change of the antiporter and consequently affects its activity.

### Computational analyses

After having experimentally characterized the Na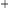/H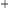 antiporter from *Yersinia pestis* we set forth to examine it computationally in order to attempt and put our results in a structural context.

#### Homology modeling

Homology modeling of membrane proteins is similar in nature to that of water soluble proteins. Furthermore, results obtained when homology modeling membrane proteins are as good as those obtained when modeling their water soluble counterparts [27]. Given that the *Yersinia pestis* NhaA shares a high identity (67%) and similarity (80%) with the *Escherichia coli* NhaA (E value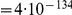), the latter was selected as a template for the homology modeling process (Figure 1). The extensive sequence resemblance between the proteins ensures a high success of the modeling process.

#### Model quality assessment

Prior to the MD simulations, the *Yersinia pestis* NhaA model structure was evaluated for its quality. The Ramachandran plot showed that 98.4% of the residues lie in allowed regions (from which 83.7% in the most favored regions). No major stereochemical clashes and bad contacts for main-chain or side-chain parameters were detected. Global analysis of the model with ProSA [28], showed a Z-Score of −1.49, implying no significant deviation from the template *Escherichia coli* NhaA structure. The Verify3D analysis [29], indicated a reasonably good sequence-to-structure agreement with an average score of 0.29. Thus, the resulted model of the homology modeling procedure (and before the MD simulations) was a relatively reliable structure.

#### Overall three-dimensional structure

**Figure 5:**
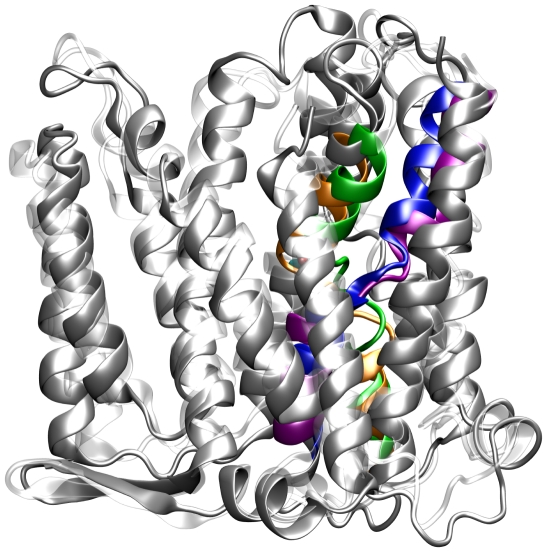
Schematic diagrams of a representative structure derived from one of the 20 ns simulations of the *Yersinia pestis* NhaA (gray) superimposed upon the x-ray structure (PDB entry 1ZCD) of the *Escherichia coli* NhaA (white). TMSs IV and XI of the *Yersinia pestis* NhaA are colored with orange and purple and these of the *Escherichia coli* with green and blue, respectively.

The two structures of the protein from both bacteria highly resemble each other, and the *Yersinia pestis* NhaA displays all the distinguishing characteristics of the *Escherichia coli* protein (see Figure 5). The *Yersinia pestis* NhaA comprises of twelve TMSs; TMSs IV (orange) and XI (purple) form the unique assembly exactly as at the *Escherichia coli* protein, the N- and C- termini are exposed to the cytoplasm (see below), a funnel opens into the cytoplasm and continues to the middle of the membrane, a shallower funnel opens to the periplasm and is separated from the cytoplasmic funnel by non-polar residues that act as a barrier, the periplasmic face of the protein is flat owing to structured loops and the cytoplasmic face is rough with flexible loops and a few helices that protrude into the cytoplasm.

Regulation of the antiporting activity of *Escherichia coli* NhaA requires a “pH sensor”, which was found to potentially include residues such as E78, E252, H253 and H256 [30]. These residues are located at the N-terminal side of the cytoplasmic funnel not only at the *Escherichia coli* template but also at the suggested model for the *Yersinia pestis* NhaA. A few key functional residues located at TMS II (D65), TMS IV (D133), TMS V (D163, D164) and TMS X (L296, G299, K300, G303) were found to play significant physiological roles at the *Escherichia coli* NhaA [7], [31], [32]. All these residues are conserved and fit exactly to the same helices at the *Yersinia pestis* NhaA model structure as well; furthermore, they maintain their orientation throughout the simulations of both proteins. Overall, the architecture of our suggested model for the *Yersinia pestis* NhaA retains the distinctive *Escherichia coli* NhaA characteristics.

**Figure 6:**
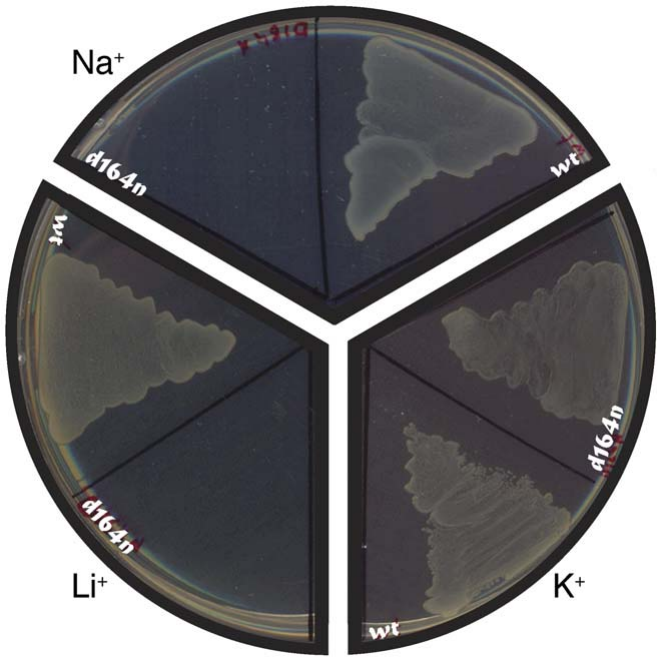
Complementation assay of the D164N mutant. *Yersinia pestis* NhaA wild-type and D164N mutant were expressed in KNabc bacterial strain that does not harbor any native antiporter [20]. Sections of plates using different cations, at 170 mM, as indicated, are presented.

To further validate the architectural similarity of the two proteins, we mutated the key residue, D164, to asparagine and tested the variant for complementation ability. The results of the complementation assay (Figure 6) show that bacteria expressing the mutant cannot grow in the presence of Na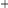 or Li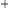. These results suggest that the mutation produces a nonviable protein, in line with the corresponding mutation in *Escherichia coli* [11].

#### Stability

MD simulations can be used as a tool to test the quality of homology modeled membrane proteins [33], [34]. In order to test the resulted model of the homology modeling procedure, we performed three independent MD simulations (each for a duration of 20 ns) of the protein when embedded in a membrane and at the presence of a physiological salt concentration. Representative structure from the MD simulations of the *Yersinia pestis* NhaA (gray), superimposed with the x-ray structure of the *Escherichia coli* NhaA (white) for comparison, are presented on Figure 5.

Several measures were employed in order to gauge the stability of the homology model-derived structure by analyzing three independent simulations. We measured the deviation of the simulated structure from the original structure, the fluctuation of the residues of the protein's model and its secondary structure.

**Figure 7:**
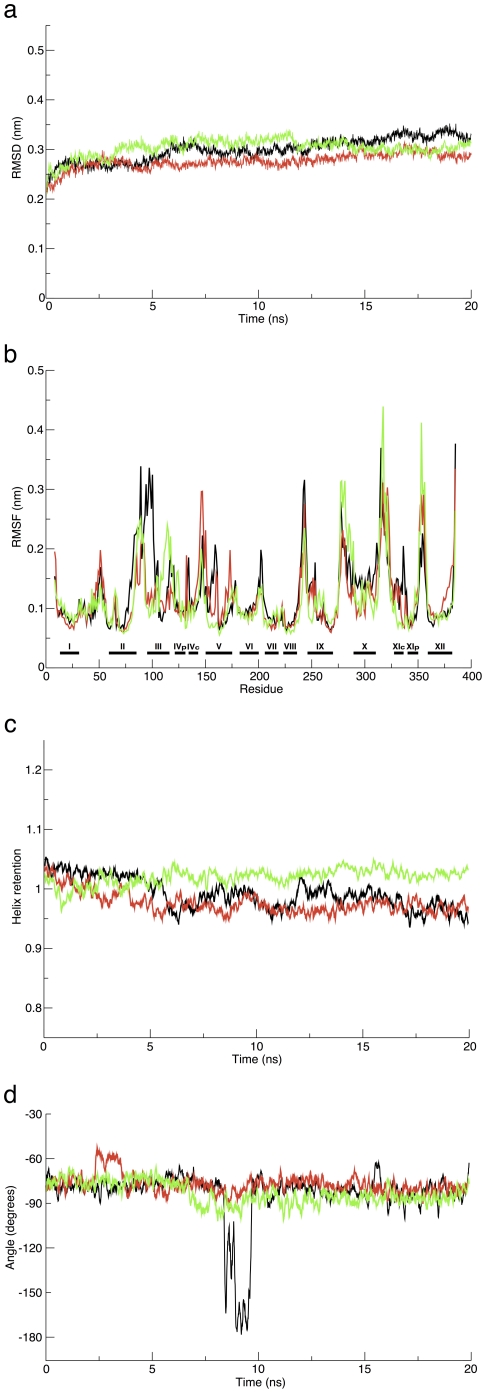
Analysis of structural parameters of the *Yersinia pestis* NhaA protein as a function of the 20 ns MD simulations. (a) RMSD for the C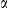 atoms; (b) RMSF, aligned by sequence, TMSs marked with horizontal bars; (c) Relative helix retention and (d) Accessibility of D164 to the vestibule's core. The different colors on each panel represent different trajectories, each represent an independent unbiased trial.

Monitoring the time evolution of the system (Figure 7 a), revealed that the protein was relatively stable. Its C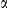 RMSD values were pretty low during the course of the simulations, whereby all simulations diverged from the initial structure by a C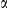 RMSD of 0.26–0.33 nm. The RMSD curve pattern implies that the structure did not substantially deviate during the simulations and were stable. This was further verified by following the root mean square fluctuation (RMSF) of the protein (Figure 7 b). Not surprisingly, the RMSF curves of the three simulations present relatively mild oscillations. Notably, the structural sections of the proteins, namely their twelve TMSs (which are marked by black horizontal bars and labeled with roman numerals at the bottom of Figure 7 b), are more rigid and confined and tend to be less flexible than other sections. Correspondingly, the proteins' unstructured sections show increased motility. Thus, at all simulations, the RMSF data indicate larger fluctuations of segments belonging to loops that connect the helices, as well as of residues located at the edges of the 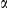-helical sections or edges of the protein. Noteworthy, all three curves are quite similar to each other which implies that in all simulations the protein undergoes similar adjustments, supporting the internal convergence of the structural features and characteristics in the three trials. Finally, the dynamics of TMS X during the simulations is not typical for helical segments, and hence it has a relatively high RMSF among all the other TMSs. Inspection of its dynamics during the simulations revealed that it bent and tilted similarly to previous findings [35].

Next, we followed the helix retention of the protein as a function of time (Figure 7 c). The helix retention of the protein was similar and stable in all simulations; at one simulation the 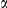-helical content slightly increased by 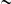 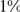 throughout the simulation time, whereas at the other two simulations marginally decreased to 97–99%. The high retention of the 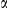-helical content throughout the simulations time at all cases implies that the protein's secondary structure elements are steady, meaning that the TMSs do not unwind.

The entry of ions from the cytoplasm to the vicinity of the TMSs IV/XI assembly is performed through a vestibule that spans from the cytoplasmic face of the protein and ends in the middle of the membrane near D164 [7]. The mode of ion binding is most probably a direct attachment to the carboxyl group of the D164 side chain, presumably at its center [12]. Hence, we examined the accessibility of D164 to the vestibule's pore (Figure 7 d) by checking the dihedral angle of its rotamer (N-C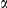-C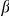-C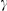) in order to observe the availability and exposure of the ion-binding site to the vestibule's space. We have found that the average D164 rotamer's dihedral angle of the *Yersinia pestis* NhaA was stable and steady throughout the simulations (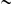
−70) enabling D164 to bind cations. Yet, at one simulation, at a short period of time, between 8.4 and 9.8 ns, the angle changed abruptly to 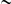
−170 due to a passing water molecule to which D164 was temporarily pointing. Nonetheless, it is evident that the most dominant rotamer is the one accessible for substrate binding from the vestibule.

### Concluding Remarks

In this study, we have biochemically characterized the Na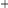/H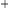 antiporter from *Yersinia pestis* and implemented homology modeling and MD simulations aimed at suggesting a working structural model. Regarding the experimental branch of this study, we prepared everted membrane vesicles and performed fluorescence quenching assays to determine its substrate specificity and monitored its pH dependence. We have shown that the antiporter is selective for Li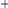 and Na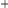 over the other tested cations, and has a higher affinity for Li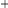 over Na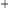. The *Yersinia pestis* NhaA activity is pH regulated; the protein is inactive at pH 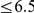 and its activation increases by 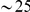-fold from pH 7 to 8. Using homology modeling, we constructed a preliminary model of the protein and evaluated its quality in three independent MD simulations to test relaxation convergence. We analyzed the overall three-dimensional structure of the protein while comparing it to the x-ray structure of the *Escherichia coli* NhaA (that was used as a template for the homology modeling process) and followed characteristics of its stability. Overall, the combined methodologies were used to gain a schematic illustrative structural model of the *Yersinia pestis* NhaA and decipher its function.

Up to now, rational design of NhaA inhibitors has not been reported, and therefore having a blueprint structure of the *Yersinia pestis* NhaA provides a basis for a functional analysis of the protein , such as *de novo* inhibitor design . An improved understanding of its function may ultimately aid design selective of blockers of NhaA, which can be useful due to the potential hazardous misuse of *Yersinia pestis* for biological warfare purposes. Considering that NHE1 (Na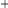/H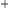 exchanger 1) and NHA2 (Na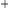/H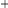 exchanger 2) [36], the human orthologs to the *Yersinia pestis* NhaA, bear no sequence similarity to *Yersinia pestis* NhaA, the latter may serve as specific potent medical target for inhibition with relatively minor undesired side-effects. However, the effect of blocking NhaA activity on the ability of *Yersinia pestis* to survive and replicate within the host cell subsequent to infection still needs to be validated, and hence its role as a potential medical target is yet to be proven. Nonetheless, due to the potential clinical importance of the *Yersinia pestis* NhaA protein (considering the lack of NhaB, NhaC or NhaD in *Yersinia pestis*) and the epidemiological need for NhaA inhibitors, we hope that our model and the biochemical characterization will be considered useful not only from a mechanistic perspective but from a pharmaceutical point of view as well.

## Materials and Methods

### Bacterial strains and plasmids

Due to biological hazard considerations, the *Yersinia pestis* NhaA gene was cloned from the closely related *Yersinia pseudotuberculosis* bacterium (given by the late Prof. Z. Zelinger, The Hebrew University of Jerusalem) using colony PCR with flanking primers. The gene was sub-cloned into a pBR322-derived plasmid regulated under NhaR [37] (given by Prof. E. Padan, The Hebrew University of Jerusalem). Using Stratagene's QuikChange site-directed mutagenesis kit (Agilent Technologies, Santa Clara, CA) we mutated the *Yersinia pseudotuberculosis* NhaA to fit the protein sequence of *Yersinia pestis* NhaA. The same kit was used to perform the D164N mutation . Plasmids bearing *Yersinia pestis* NhaA and an empty plasmid for control were transformed into *Escherichia coli* KNabc strain (TG1 derivative, 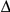
nhaA 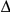
nhaB 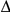
chaA [20]) which is strongly inhibited by NaCl and LiCl. Plasmid amplification was done in *Escherichia coli* DH5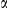 cells. Growth media was Lysogeny Broth (LB) [38]. Antibiotics concentration was 100 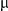
g/ml ampicillin.

### Vesicles preparation and fluorescence quenching

Everted membranes were produced using the technique introduced by Rosen and Tsuchiya [39] with the following steps: lysis buffer used contained 21% sucrose, 15 mM of Tris/HCl buffer at pH 7.5 and 150 mM of choline-chloride. Bacteria were grown overnight in LB medium, washed three times in lysis buffer, suspended in 5 ml/gr and broken once in a French Press at 900 psi (valve pressure). Broken bacteria solution was centrifuged at 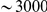 g for 20 minutes and the supernatant was further centrifuged at 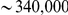 g for 20 minutes. The final pellet, containing the vesicles, was resuspended in lysis buffer with 1 ml/gr of original dry bacteria, and frozen in liquid nitrogen.

NhaA activity was measured by the quinacrine fluorescence quenching method [21], [22], using lysis buffer and 2.4 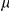
M of Acridine Orange. Succinic acid (0.6 mM) was used to energize the vesicles. 100% quenching was defined as the difference in fluorescence between the level prior to addition of succinic acid and the level after a steady state was achieved. NhaA activation level was defined as the fraction of dequenching at steady state after substrate addition, relative to 100% quenching previously defined. Fluorescence readings were obtained by excitation at 366 nm and emission at 531 nm using the Perkin-Elmer LS-45 luminescence spectrophotometer.

Kinetic analysis was done using the fluorescence quenching results. Activations under different ion concentrations were plotted together using a Michaelis-Menten simple enzyme kinetic model [40]. Regression was performed using the non-linear least sum of squares technique. Statistical data were obtained using the bootstrapping method on the entire dataset, consisting of 3 repeats for the data points indicated and several more data points randomly distributed. The bootstrapping process was done by running a 1000 cycles and its convergence was evaluated using standard statistical procedures.

### Homology modeling

The template used to model NhaA from *Yersinia pestis* (Entrez accession code NP_994981) was NhaA from *Escherichia coli* (PDB entry 1ZCD, chain A). The amino acid sequences of the NhaA from both bacteria were aligned using BLAST [41], [42] with the blastp algorithm. Homology models were generated with Modeller 9.4 [43]. The model with the best score was assessed for its quality with respect to its energy and stereochemical geometry using Procheck 3.5.4 [44], [45], ProSA-Web [28], [46] and Verify3D [29].

### System set-up

The model with the best score was inserted into a pre-equilibrated 1-palmitoyl-2-oleoyl-sn-glycero-3-phosphoethanolamine (POPE) bilayer (adopted and modified from [47]). The insertion was done so that the protein's rough axis is perpendicular to the membrane surface plane, and all colliding lipids and water molecules, within 2 Å of the protein, were removed. Extending, outside membrane, protein edge terminals were deleted. K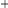 and Cl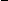 ions were added to a final concentration of 0.1 M, with a system net-charge of zero, randomly distributed. Finally, the system was subjected to rigorous energy minimization using the steepest descent algorithm and tolerance of 1000 kJ

mol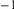 
nm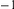, followed by a minimization using the conjugated gradient algorithm with a sequential decreasing convergence from 100 to 10 kJ

mol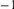 
nm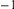. Then, an equilibration stage under positional restraints using a harmonic force constant was conducted. The equilibration procedure began with a force constant of k = 1000 kJ

mol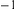 
nm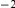 for 100 ps, then a force constant of k = 500 kJ

mol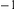 
nm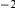 for 100 ps, and another 100 ps of an unrestrained MD run. This allowed the lipids and water to pack more tightly around the protein, and enable the protein gradual relaxation in the membrane. After the positional restraint equilibration, the systems were submitted for unbiased MD runs.

### MD details

The systems were subjected to three trials of a 20 ns MD simulation each, in order to test their convergence. The simulations were conducted using version 3.3.1 of the GROMACS package [48], [49], employing an extended version of the GROMOS53a6 force field [50]. The simulations were conducted using the LINCS algorithm [51] to constrain bond lengths and angles of hydrogen atoms, allowing a time step of 2 fs. Simulations were run using Berendsen temperature coupling at 310 K employing a coupling constant of 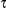 = 0.1 ps. Pressure was kept constant at 1 bar by applying semi-isotropic coupling with a coupling constant of 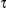 = 1 ps, differentiating the *z*-axis (the membrane normal). A cutoff of 1.2 nm was used for van der Waals interactions; long range electrostatic interactions were computed using the PME algorithm [52].

### Visualization and analysis

The simulations were visualized with the Visual Molecular Dynamics (VMD) program [53]. The analyses were conducted using in-house VMD Tcl scripts, in-house purpose written perl scripts, and the GROMACS analysis package tools.

## Supporting Information

Figure S1
**Basal response of everted membrane vesicles in the acridine orange fluorescence dequenching.** The bacteria do not contain any Na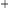/H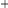 antiporter. For details see figure 3 in the main text.(TIF)
