# Supplementary figures and images for: Characterization of the Na+/H+ Antiporter from Yersinia pestis
Source: PLoS One. 2011 Nov 15;6(11):e26115. doi: 10.1371/journal.pone.0026115 (PMC3216949; doi:10.1371/journal.pone.0026115)

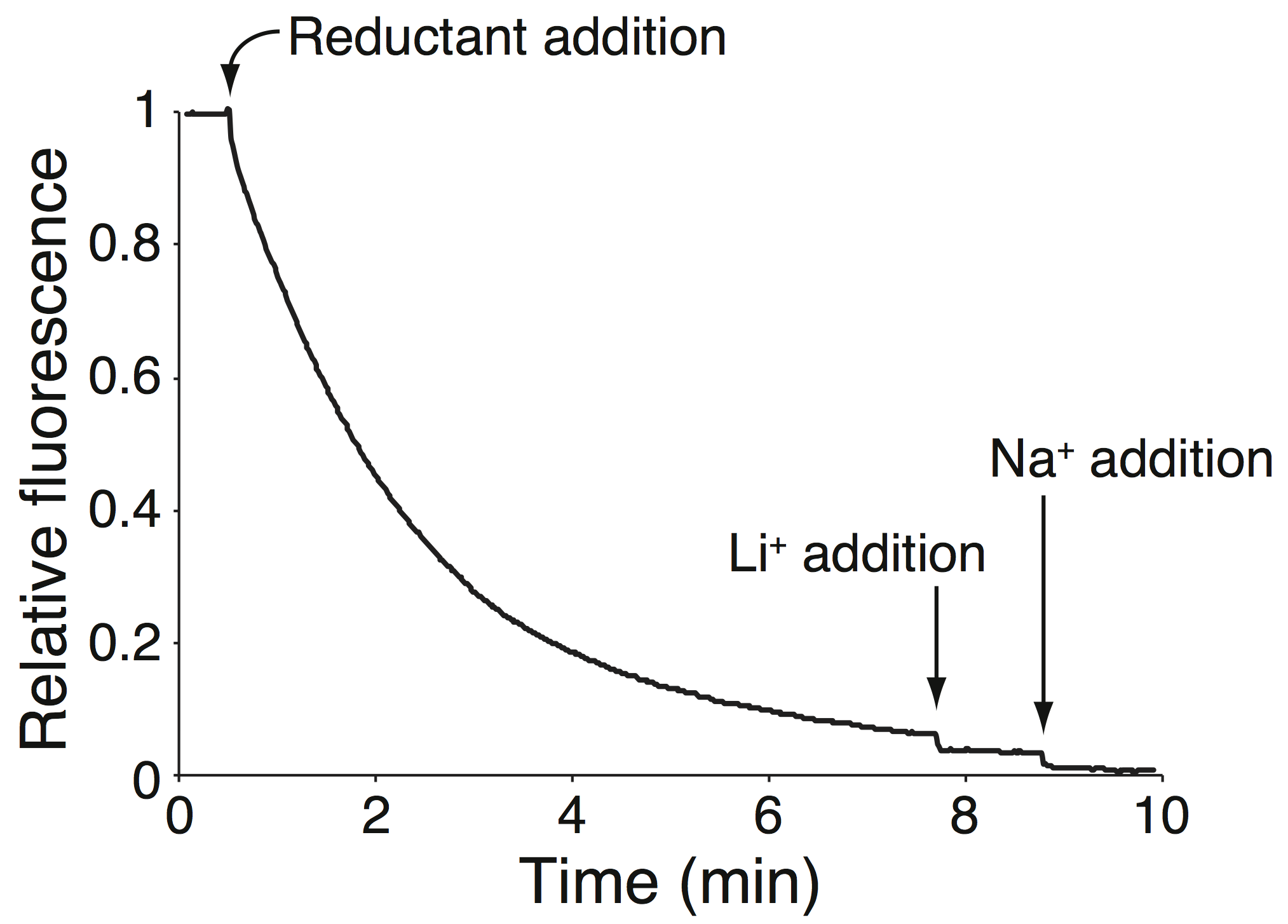

Supplement: Figure S1 — Basal response of everted membrane vesicles in the acridine orange fluorescence dequenching. The bacteria do not contain any Na/H antiporter. For details see figure 3 in the main text. (TIF) [file pone.0026115.s001.tif]
